# Telephone follow-up by clinical pharmacists can improve treatment outcomes in patients with peptic ulcers: A prospective randomized study

**DOI:** 10.1097/MD.0000000000031150

**Published:** 2022-10-21

**Authors:** Aibin Weng, Xiaoyue Su, Changqing Yang, Bijin Zheng, Lili Zheng, Chenxing Jian, Jianying Fang

**Affiliations:** a Department of Pharmacy, Affiliated Hospital of Putian University, Putian, China; b Department of Pharmacy, Putian Medical District, No.900 Hospital of Joint Logistics Support Force, Putian, China; c Department of Digestive Internal Medicine, Affiliated Hospital of Putian University, Putian, China; d Department of Minimally Invasive Surgery, Affiliated Hospital of Putian University, Putian, China.

**Keywords:** clinical pharmaceutical care, compliance, peptic ulcer, telephone follow-up

## Abstract

The purpose of this study was to investigate the effect of pharmaceutical care by clinical pharmacists through telephone follow-up on the treatment outcomes in patients with peptic ulcers who had been discharged from the hospital.

**Methods:**

A total of 120 patients with peptic ulcers discharged from the hospital were randomly divided into an intervention group and a control group, with 60 patients in each group. The patients in the two groups received different services.

**Results:**

A total of 108 patients with peptic ulcers were enrolled in this study, including 53 in the intervention group and 55 in the control group. This study showed that the *Helicobacter pylori* eradication rate (19/23, 82.61% vs 13/29, 44.83%), awareness of peptic ulcer disease, medication compliance, diet compliance, and life compliance in the patients in the intervention group were higher than those in the patients in the control group. The *H pylori* eradication group had higher follow-up scores than the noneradication group. Sex and body mass index (BMI) did not affect the results in either group, but age did. In the control group, younger patients scored higher than older patients, whereas in the intervention group, this difference disappeared for diet compliance and life compliance, and significant differences remained for awareness of basic knowledge regarding peptic ulcer (AOKPU) and medication compliance.

**Conclusion:**

As a form of clinical pharmaceutical care, telephone follow-up by clinical pharmacists is recommended for discharged patients with peptic ulcers because it can improve treatment outcomes after discharge.

## 1. Introduction

Peptic ulcer is a chronic disease; its development and prognosis are closely associated with patient awareness of the disease and treatment compliance, and family treatment after discharge is associated with the treatment effect. A study by Thomas Mooe et al^[1]^ showed that nurse-based secondary preventive follow-up by telephone reduced the recurrence of cardiovascular events. Several studies on hypertension, diabetes, heart failure, rheumatoid arthritis, and cancer have also demonstrated the benefit of telephone follow-ups in these patients.^[2–6]^ Therefore, pharmaceutical intervention care plays an important role in the treatment process. A new question then arises: Is this type of pharmaceutical follow-up effective in patients with peptic ulcer? Based on this question, we designed this prospective randomized study. The objective of this study was to explore the influence of pharmaceutical follow-up in discharged peptic ulcer patients to understand whether clinical pharmaceutical follow-up services can provide discharged patients with a deeper understanding of peptic ulcer disease, strengthen the treatment compliance of discharged patients, and thus improve the treatment effect and quality of life of these patients.

## 2. Methods

### 2.1. General demographic data

A total of 120 patients with peptic ulcer admitted to the Affiliated Hospital of Putian University from August 2018 to March 2019 were included in this study.

### 2.2. Design of the study

Eligible patients were selected from a cohort of discharged peptic ulcer patients and randomly divided into 2 groups: the control group and the intervention group, with 60 patients in each group (Flow Diagram). The patients in the control group received routine services at discharge, while the patients in the intervention group received routine services at discharge followed by clinical pharmacist education and regular telephone follow-ups after discharge. The telephone follow-ups were conducted by a trained clinical pharmacist at 1 week and 3 weeks after discharge and at the end of the course of medication. During the primary follow-up, clinical pharmacists introduced basic knowledge about peptic ulcer to the patients to relieve any psychological pressure experienced by patients. The pharmacist also instructed the patients on medication use, diet, and exercise and informed the patients of the importance of regular follow-up.

The inclusion criteria were as follows: 18 to 70 years of age; digestive ulcer diagnosed by electronic gastroscopy; complete hospitalization records; and voluntary cooperation in the study. The exclusion criteria were as follows: age younger than 18 years or older than 70 years; severe underlying diseases; pregnancy or lactation; severe complications or allergies to drugs during treatment; and communication difficulties.

### 2.3. Evaluation indicators

#### 2.3.1. Evaluation index of the knowledge rate of ulcer basic knowledge.

Awareness of basic knowledge regarding peptic ulcer (AOKPU) was analyzed according to the proportion of people with relevant knowledge. Patients AOKPU was evaluated using three questions: (Question A1) Do you know why you are in the hospital?, (Question A2) Do you know your course of treatment? and (Question A3) Do you know your main medication? Those who answered these three questions accurately were considered to have complete knowledge; those who answered 2 questions accurately were considered to have partial knowledge; and those who answered 1 question accurately or did not know any of the answers were considered to have no knowledge.

#### 2.3.2. Evaluation indicators of treatment compliance.

The Morisky-Green (MG) scale^[7]^ was used to measure medication compliance in patients with peptic ulcer disease. The scale included four questions: (Question B1) Have you ever missed your medication?, (Question B2) Have you ever not taken your medicine according to the doctor’s instructions (time, dosage, etc)?, (Question B3) Have you discontinued your medication because your symptoms have lessened or because you feel no obvious symptoms?, and (Question B4) Have you stopped taking your medication because of worsening symptoms or adverse reactions? An answer of “no” was scored as 1, and “yes” was scored as 0. According to the MG scale and a large number of references reporting diet and life compliance issues, patient compliance was measured based on the following four dietary compliance questions and four life compliance questions: (Question C1) Do you eat regularly?, (Question C2) Do you consume a soft diet?, (Question C3) Do you avoid indigestible and irritating foods?, (Question C4) Have you ever given up smoking or drinking?, (Question D1) Do you have regular living habits?, (Question D2) Do you have adequate sleep quality?, (Question D3) Do you engage in proper low-intensity exercise?, and (Question D4) Do you have a positive and balanced mood? An answer of “no” was scored as 1, and “yes” was scored as 0.

### 2.4. Clinical treatment plan

*Helicobacter pylori*-negative patients received PPI + mucosal protector therapy, and *H pylori*-positive patients received quadruple therapy (PPI + bismuth + 2 antibiotics).

### 2.5. *H pylori* test

*H pylori* test results were recorded before admission and at one month after treatment. Patients were advised to avoid oral antibiotics after treatment until reexamination.

### 2.6. Statistical method

The primary endpoint was the effect of telephone follow-up on AOKPU, medication compliance, diet compliance, and life compliance. Secondary endpoints were the impacts of baseline data on AOKPU, medication compliance, diet compliance, and life compliance. SPSS 20.0 software was used for data analysis, and chi-square tests were used to analyze differences in baseline data and outcomes between the 2 groups.

## 3. Results

A total of 120 patients with peptic ulcer disease were included in this study, of which 108 patients were assessed, 8 were lost to follow-up, and 4 withdrew from the study. As shown in Table 1, there was no significant difference in the demographic characteristics between the two groups of patients (*P* > .05). As shown in Table 2, sex and body mass index (BMI) did not affect the results in either group, but age did. In the control group, younger patients scored higher than older patients for all factors (AOKPU 2.22 ± 0.58 vs 1.54 ± 0.64, *P* < .001; medication compliance 3.52 ± 0.64 vs 2.71 ± 0.85, *P* < .001; diet compliance 3.85 ± 0.46 vs 3.54 ± 0.51, *P* = .019; life compliance 3.93 ± 0.27 vs 3.71 ± 0.46. *P* = .042), whereas in the intervention group, this difference disappeared for diet compliance and life compliance (diet compliance 4.00 ± 0.00 vs 3.93 ± 0.26, *P* = .161; life compliance 4.00 ± 0.00 vs 3.97 ± 0.19, *P* = .368) and significant differences remained for AOKPU and medication compliance (AOKPU 3.00 ± 0.00 vs 2.69 ± 0.47, *P* = .001; medication compliance 4.00 ± 0.00 vs 3.72 ± 0.59, *P* = .018).

**Table 1:** Comparison of basic data of the 2 groups of patients.

	Intervention group (n = 53)	Control group (n = 55)	*P*
Sex			.45
Male	41 (77.36%)	39 (70.91%)	
Female	12 (22.64%)	16 (29.09%)	
Age	45.08 ± 14.28	46.11 ± 16.27	.73
BMI	23.20 ± 4.04	23.94 ± 3.24	.30
Smoking history			.12
Yes	0(0.00)	4(7.3)	
No	53(100.00)	51(92.7)	
Payment			.94
Residents’ insurance	40 (75.47%)	41 (74.55%)	
Workers’ insurance	10 (18.87%)	10 (18.18%)	
Self-pay	3 (5.66%)	4 (7.27%)	
Ulcer type			.64
Gastric ulcer	7(13.21%)	11(20.00%)	
Duodenal ulcer	41(77.36%)	39(70.91%)	
Compound ulcer	5(9.43%)	5(9.09%)	
Presentation			.64
Melena	28(52.83%)	27(49.09%)	
Hematemesis and melena	8(15.09%)	7(12.73%)	
Epigastric pain	7(13.21%)	10(18.18%)	
Hematemesis	2(3.77%)	5(9.09%)	
Others*	8(15.09%)	6(10.9%)	

BMI: body mass index.

* In the intervention group, there were 3 cases of colonic polyps, 1 case of colonic polyps and posturinal bleeding, 1 case of coffee-colored contents and melea, 1 case of lower abdominal pain and melea, 1 case of lower abdominal discomfort, and 1 case of right lower abdominal pain. In the control group, there was 1 case of colon polyps; 1 case of upper abdominal pain, vomiting and melenia; 1 case of abnormal defecation; 1 case of acid reflux, nausea and vomiting; 1 case of abdominal pain and diarrhea; and 1 case of abdominal pain and vomiting.

**Table 2:** The effects of different baseline factors on the outcomes.

	AOKPU	Medication compliance	Diet compliance	Lifestyle compliance
Intervention group				
Sex				
Male (n = 41)	2.83 ± 0.38	3.83 ± 0.50	3.95 ± 0.22	3.98 ± 0.16
Female (n = 12)	2.83 ± 0.39	3.92 ± 0.29	4.00 ± 0.00	4.00 ± 0.00
	*P* = .974	*P* = .564	*P* = .445	*P* = .593
Age				
<44 yr (n = 24)	3.00 ± 0.00	4.00 ± 0.00	4.00 ± 0.00	4.00 ± 0.00
≥44 yr (n = 29)	2.69 ± 0.47	3.72 ± 0.59	3.93 ± 0.26	3.97 ± 0.19
	*P* = .001	*P* = .018	*P* = .161	*P* = .368
BMI				
<23.03 (n = 26)	2.88 ± 0.33	3.96 ± 0.20	4.00 ± 0.00	4.00 ± 0.00
≥23.03 (n = 27)	2.78 ± 0.42	3.74 ± 0.59	3.93 ± 0.27	3.96 ± 0.19
	*P* = .307	*P* = .077	*P* = .161	*P* = .327
Control group				
Sex				
Male (n = 39)	1.87 ± 0.73	3.13 ± 0.89	3.64 ± 0.54	3.79 ± 0.41
Female (n = 16)	1.88 ± 0.62	3.06 ± 0.77	3.81 ± 0.40	3.88 ± 0.34
	*P* = .988	*P* = .798	*P* = .204	*P* = .493
Age				
<44 yr (n = 27)	2.22 ± 0.58	3.52 ± 0.64	3.85 ± 0.46	3.93 ± 0.27
≥44 yr (n = 28)	1.54 ± 0.64	2.71 ± 0.85	3.54 ± 0.51	3.71 ± 0.46
	*P* < .001	*P* < .001	*P* = .019	*P* = .042
BMI				
<23.03 (n = 27)	1.74 ± 0.59	3.00 ± 0.83	3.63 ± 0.57	3.74 ± 0.45
≥23.03 (n = 28)	2.00 ± 0.77	3.21 ± 0.88	3.75 ± 0.44	3.89 ± 0.32
	*P* = .169	*P* = .357	*P* = .381	*P* = .152

AOKPU = awareness of basic knowledge regarding peptic ulcer, BMI = body mass index.

*H pylori* eradication rates (19/23, 82.61% vs 13/29, 44.83%), AOKPU (2.83 ± 0.38 vs 1.87 ± 0.70, *P* < .001), medication compliance (3.85 ± 0.46 vs 3.11 ± 0.85, *P* < .001), diet compliance (3.96 ± 0.19 vs 3.69 ± 0.50, *P* < .001), and life compliance (3.98 ± 0.14 vs 3.82 ± 0.39, *P* = .005) data are shown in Table 3. The intervention group had significantly better outcomes than the control group. Among *H pylori*-positive patients, those with negative conversion showed higher follow-up scores for AOKPU (2.44 ± 0.67 vs 1.60 ± 0.75, *P* < .001), medication compliance (3.63 ± 0.66 vs 2.80 ± 0.95, *P* = .002), and diet compliance (3.91 ± 0.30 vs 3.40 ± 0.60, *P* = .002) than those who did not eradicate the disease. However, there was no significant difference in life compliance (3.91 ± 0.30 vs 3.70 ± 0.47, *P* = .090), as shown in Table 4.

**Table 3:** Comparison of intervention outcomes between the 2 groups.

	Intervention group (n = 53)	Control group (n = 55)	*P*
H.Pylory			.332
Positive	23(43.40%)	30(56.60%)	
Negative	30(56.60%)	26(47.27%)	
Eradication of H.Pylory	19/23(82.61%)	13/29(44.83%)	.005
AOKPU	2.83 ± 0.38	1.87 ± 0.70	<.001
Medication compliance	3.85 ± 0.46	3.11 ± 0.85	<.001
Diet compliance	3.96 ± 0.19	3.69 ± 0.51	<.001
Life compliance	3.98 ± 0.14	3.82 ± 0.39	.005

AOKPU = awareness of basic knowledge regarding peptic ulcer.

**Table 4:** Scores of all positive patients.

	Turned negative (n = 32)	Not turned negative (n = 20)	*P*
AOKPU	2.44 ± 0.67	1.60 ± 0.75	<.001
Medication compliance	3.63 ± 0.66	2.80 ± 0.95	.002
Diet compliance	3.91 ± 0.30	3.40 ± 0.60	.002
Life compliance	3.91 ± 0.30	3.70 ± 0.47	.090

AOKPU = awareness of basic knowledge regarding peptic ulcer.

## 4. Discussion

The ratio of male to female patients in this study was approximately 2.86:1, which is similar to those previous studies, possibly because males have more negative habits than females, such as smoking, alcohol consumption, and irregular work and rest, and they experience higher social pressure.^[8–10]^

The higher a patient’s cognition of the disease is, the higher their self-adjustment ability and self-efficacy are. Guided intervention can improve a patient’s cognition of their disease and compliance with treatment.^[6,11]^ In this study, after discharge education and post-discharge guidance and supervision by clinical pharmacists, the patients in the intervention group had significantly higher AOKPU scores than the control group. However, the patients in the control group received only routine services, with no discharge guidance or regular follow-up; therefore, these patients did not have a comprehensive understanding of ulcers and were not advised about precautions during the treatment process. Among the *H pylori*-positive patients, the intervention group showed a higher rate of negative conversion than the control group (19/23, 82.61% vs 13/29, 44.83%). One possible reason is that the intervention group had higher medication compliance. After discharge, most of the patients in the intervention group were able to adhere to the correct drug regimen throughout the whole process, while those in the control group were more likely to miss their oral medications or take their oral mediations irregularly. The main issue that may have affected medication compliance in patients in the control group was a nontimely follow-up visit. Poor adherence to the drug regimen was largely due to lack of knowledge about the course of peptic ulcer treatment; patients who are discharged from the hospital often think their disease has been cured and may only take their prescribed medications for a few days after leaving the hospital. Second, due to the long disease treatment course, patients must take their medications for a long time; thus, missing a dose or otherwise mismanaging the drug regimen is common. In addition, most of the patients were older, and older age was associated with low medication compliance. The current study also found that some drugs that need to be stored in refrigerators, such as esomeprazole enteric-soluble capsules and *Bifidobacterium lactobacillus* triplex tablets, were not stored correctly. In such cases, the patients may not have understood or may not have heard the pharmacist’s instructions, or they may have forgotten the instructions by the time they arrived home. Therefore, clinical pharmacists can provide clinical pharmaceutical care to discharged patients and effectively improve the clinical outcomes of discharged patients with peptic ulcer disease.

Compliance among patients with peptic ulcer disease refers to not only following the instructions and suggestions of medical staff for treatment during hospitalization but also adhering to the medical advice for treatment after discharge. The results of this study showed that diet compliance in the intervention group was better than that in the control group (3.91 ± 0.30 vs 3.40 ± 0.60, *P* = .002). Most of the patients in the intervention group were able to adhere to guidance about quitting drinking, maintaining a regular light diet, and avoiding indigestible food. In contrast, patients in the control group mismanaged their drug regimen after discharge, stopped taking drugs when their symptoms improved, returned to a normal diet without dietary restrictions after discharge, and did not seek regular care. Regarding life compliance, including quitting smoking, maintaining a reasonable work and rest schedule, avoiding overwork, and maintaining a happy mood, there were no significant differences between the two groups (3.91 ± 0.30 vs 3.70 ± 0.47, *P* = .090). It is possible that these patients were hospitalized with more serious symptoms, and it was easier to accept changes in living habits, which are obviously different from drug compliance, because more people may have certain doubts about the continued use of drugs after symptoms are relieved.

Analysis of the impacts of baseline factors on the results seemed to indicate that the intervention had a positive effect on dietary compliance and life compliance in older patients. It is possible that older patients may have difficulty accessing information about peptic ulcer disease, and this intervention fills this knowledge gap.

**Figure 1.**
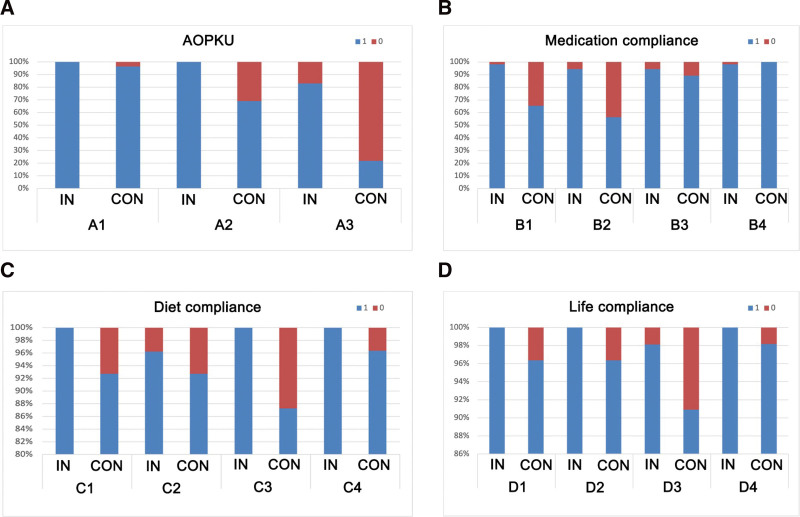
The responses of patients to various follow-up items. The meanings of A1-3, B1-4, C1-4 and D1-4 refer to the follow-up questions in the Methods section. Con = control group, In = intervention group.

Figure 1 shows the responses of patients to the follow-up items. Regarding AOKPU, patients in the control group had significant knowledge deficits in treatment and medication. In terms of medication adherence, patients in the control group were more likely to miss or not take their medication as prescribed. Although precise statistical analysis is not possible, this may be related to the low educational level of some patients. Regarding dietary compliance, indigestible and irritating foods, which are often components of local food traditions and are associated with misconceptions about food, are a prominent problem. For example, the peanut is a traditional food, but its associated harm has not received adequate attention. Finally, in terms of life compliance, the overall difference was not significant, although the control group had slightly lower rates of low-intensity exercise. These results can guide topics to be addressed in future follow-up work.

In the statistical analysis of all positive patients, patients with negative conversion received higher follow-up scores, as shown in Table 3. This also explains the correlation between *H pylori* negative conversion and clinical pharmaceutical follow-up. Telephone follow-up is a widely used follow-up method^[12]^ that enables discharged patients to receive health guidance and medical services related to their disease at home and reduces the recurrence of cardiovascular events during long-term follow-up.^[1]^ It is well known that Chinese doctors are often overwhelmed by overwork and do not have the time and energy to carefully instruct each patient on medication use. Clinical pharmacists can compensate for this deficiency, which can not only reduce the burden on doctors but also solve the problems of patients in the follow-up process and meet the needs of patients.

There are some limitations of this study. First, the small number of patients included in the study may result in bias. Second, the long-term effects of this pharmaceutical intervention are unknown, and we look forward to continuing to study its long-term effects in the future.

## 5. Conclusion

This study showed that as a form of clinical pharmaceutical care, telephone follow-up by clinical pharmacists for discharged patients with peptic ulcer disease improved treatment compliance and treatment outcomes after discharge; therefore, this intervention is recommended.

## Author contributions

**Funding acquisition:** Jianying Fang.

**Project administration:** Aibin Weng, Chenxing Jian, Jianying Fang.

**Supervision:** Aibin Weng, Jianying Fang.

**Validation:** Aibin Weng, Xiaoyue Su, Jianying Fang.

**Writing – original draft:** Aibin Weng, Xiaoyue Su, Changqing Yang, Bijin Zheng, Lili Zheng, Chenxing Jian.

**Writing – review & editing:** Aibin Weng, Chenxing Jian, Jianying Fang.
